# Perceived coaches’ health promotion activity, maintenance of participation in sports, and lifestyle habits among emerging adults: a four-year follow-up study

**DOI:** 10.1080/07853890.2024.2321327

**Published:** 2024-04-24

**Authors:** Katja Rinta-Antila, Pasi Koski, Tuula Aira, Olli J. Heinonen, Raija Korpelainen, Jari Parkkari, Kai Savonen, Kerttu Toivo, Arja Uusitalo, Maarit Valtonen, Tommi Vasankari, Jari Villberg, Sami Kokko

**Affiliations:** a Faculty of Sport and Health Sciences, Research Centre for Health Promotion, University of Jyväskylä, Jyväskylä, Finland; b Department of Teacher Education, University of Turku, Rauma, Finland; c Paavo Nurmi Centre & Unit for Health and Physical Activity, University of Turku, Turku, Finland; d Medical Research Center (MRC), University of Oulu and University Hospital of Oulu, Oulu, Finland; e Department of Sports and Exercise Medicine, Oulu Deaconess Institute Foundation sr., Oulu, Finland; f Research Unit of Population Health, University of Oulu, Oulu, Finland; g Tampere Research Center of Sports Medicine, Tampere, Finland; h Institute of Public Health and Clinical Nutrition, University of Eastern Finland, Kuopio, Finland; i Kuopio Research Institute of Exercise Medicine, Kuopio, Finland; j Clinic for Sports and Exercise Medicine, Foundation for Sports and Exercise Medicine, Helsinki, Finland; k Department of Sports and Exercise Medicine, Clinicum, University of Helsinki, Helsinki, Finland; l Research Institute for Olympic Sports, Jyväskylä, Finland; m UKK Institute of Health Promotion Research, Tampere, Finland; n Faculty of Medicine and Health Technology, Tampere University, Tampere, Finland

**Keywords:** Cohort study, settings-based health promotion, health behavior, sports club

## Abstract

**Objectives:**

This study focused on how adolescents’ perceptions of coaches’ health promotion activity predict maintained participation and dropout in organized sports in emerging adulthood. In addition, differences in lifestyle habits between maintainers, dropouts, and nonparticipants in organized sports were explored.

**Materials and Methods:**

Overall, 616 adolescents reported organized sports participation in the Finnish Health Promoting Sports Club (FHPSC) study at ages 15 and 19. Of these, 323 reported coach’s health promotion activity on health topics at the age of 15. An index of a coach’s general health promotion activity was formed. At age 19, all study participants reported their lifestyle habits.

**Results:**

Among males, those who had perceived coaches’ general health promotion activity as frequent were more likely to be maintainers than dropouts (48.6% vs. 20.0%) (*p* = .002). Among females, there was no significant difference (32.0% vs. 28.4%). Logistic regression analysis adjusted for gender showed that perceiving coach’s general health promotion activity as frequent increased the odds of being a maintainer rather than a dropout. Moreover, maintainers had higher odds of having healthy lifestyle habits when compared to nonparticipants (related to physical activity; sleep; fruit and vegetable consumption; and cigarette use) or dropouts (related to physical activity; and cigarette use). In addition, dropouts had higher odds of having healthy lifestyle habits than nonparticipants (related to sleep; and cigarette use).

**Conclusions:**

Perceiving coaches’ health promotion activity as frequent was related to maintained participation in organized sports among males. Maintainers were more likely to have more healthy lifestyle habits than nonparticipants and dropouts. There is a need to invest in coaches’ health promotion activity when it is infrequent. A more detailed understanding is needed of coaches’ health promotion activity that supports both maintained participation in sports, especially among females, and healthy lifestyle habits in emerging adulthood.

## KEY MESSAGE

There is a need to invest in coaches’ health promotion activity when it is infrequent in order to support adolescents’ continued participation in organized sports and healthy lifestyle habits in emerging adulthood.

## Introduction

Sports clubs, as settings of organized sports, informal education, and voluntary participation, have been acknowledged as potential settings for health promotion [1–4]. Participation in club sports is popular among children and adolescents [5, 6], and it has various physical (e.g. fitness) [7], psychological, and social (e.g. self-esteem, social interaction) [8] health benefits despite some disadvantages (e.g. injuries) [9]. Health promotion in sports clubs is more than increasing participants’ physical activity via sports activities. This includes, for example, creating healthy environments for sports or dealing with various health issues within sports activities [10, 11]. This benefits both sports and public health.

Previous cross-sectional research has identified sports clubs’ positive health promotion orientation [11, 12], which may lead to higher level of health promotion guidance activity [13]. This, in turn, may support the participants’ performance and development in sports [3]. Research on *coaches’ health promotion activity* has shown that coaches have estimated themselves as more active than sports participants have perceived [14]. Both coaches and sports participants have reported that from various health topics, coaches most often discuss sleep, injury prevention, and training when ill [14]. In addition, promotion of a healthy lifestyle by coaches is associated with sports participants’ enjoyment in sports [15, 16] and perceived health among adults [16]. Moreover, promoting fair play is associated with a decreased intention to drop out [15], increased enjoyment, and better perceived health among adults [16].

Longitudinal evidence is scarce. It shows how adolescents’ perceptions of sports club’s health promotion policies and practices change longitudinally and that policies and practices, for example, related to the friendliness of coaches and injury prevention, may influence sports club participartion [17]. This is important knowledge because injuries are common among organized sports participants; at least one acute injury in the past year was reported by 44% of 14 to 16 year-old [9] and by 33% of 16 to 20 year-old [18] organized sports participants. Moreover together with illness, injuries are a significant reason for dropout in organized sports for 52% of female and 45% of male dropouts aged 15 to 19 years [19]. However, to date longitudinal research on how coaches’ health promotion activity relates to maintained participation and dropout in organized sports in emerging adulthood [20], including female sports participants, is lacking [14, 16, 21].

Adolescent organized sports participants have been shown to be more likely to have *healthy lifestyle habits* (e.g. related to physical activity, nutrition, and substance use) than nonparticipants in adolescence in Norway and Finland [22–24] as well as in adulthood in Finland [25]. However, studies have shown that unhealthy lifestyle habits are common among organized sports participants. For example, about 80% of female and 70% of male organized sports participants aged 15 in Finland did not fill the recommendations for physical activity (60 min of moderate to vigorous physical activity/day) [26, 27] or sleep (at least 9 h/night) on schooldays [26, 28]. Moreover, 40–53% of female and 66–75% of male organized sports participants did not eat vegetables and fruits daily [29]. In addition, 74% of Finnish 16–20-year-old active sports club participants were not physically active enough [24]. Longitudinal research from the United States shows that substance use at age 19–22 was more likely among those who had participated in organized sports at age 18 than among nonparticipants [30]. Moreover, 40% of organized sports participants dropped out of club sports by emerging adulthood [19, 31]. However, research is mainly cross-sectional, and longitudinal studies on how changes in participation in organized sports are related to lifestyle habits in emerging adulthood are scarce.

This longitudinal study aimed firstly to explore adolescents’ perceptions of coaches’ health promotion activity and their association with the maintenance of participation in organized sports in emerging adulthood. Secondly, differences in lifestyle habits between maintainers, dropouts, and nonparticipants in organized sports were explored.

In this study, sports clubs refer to the independent organizations of the third sector typical in Finland and other Nordic as well as some European countries, mostly working on a voluntary basis, i.e. voluntary associations, providing sports for voluntary participants, having mostly voluntary but also paid coaches, using the sport facilities of the municipality or their own, charging for members and participation, and possibly receiving state and municipality subsidies [32].

## Materials and methods

### Ethics

The study was approved by the Ethics Committee of the Healthcare District of Central Finland in 2012 and 2016 (record numbers 23U/2012 and 2016). Written informed consent was obtained from the participants, and when participants were under 18 years of age, from their guardians.

### Sample

This longitudinal study was based on the health behavior questionnaire data of the Finnish Health Promoting Sports Club (FHPSC) study (see the study protocol article) [33]. The adolescents were recruited *via* sports clubs (organized sports participants) and schools (organized sports participants or nonparticipants) [33]. Double answers were combined, resulting in 2149 study participants at baseline (2013–2014). Of these, 619 adolescents (of which 382 reported being females and 227 males) aged 15 (mean age 15.5, SD 0.6) participated again at age 19 in the follow-up study (2017–2018). Of these, 191 were *maintainers* (i.e. sports participants at both time points), 255 *dropouts* (i.e. sports participants at baseline, but sport nonparticipants at follow-up), 170 *nonparticipants* (i.e. sports nonparticipants at both time points), and 3 *joiners* (i.e. sports nonparticipants in baseline, but sports participants at follow-up) at age 19 as a result of a previous FHPSC study [19]. Due to a small number, joiners were excluded from the analyses. All study participants answered questions related to lifestyle habits at the age of 19 years. Only organized sports participants recruited *via* sports clubs (*n* = 323) were asked to report their perceptions of coaches’ health promotion activity on various health topics at age 15 (Table 1).

**Table 1.** Study sample.

Baseline study participants recruited from sports clubs and schools	Follow-up study participants	Organized sports participation patterns	Total sample of study participants recruited from sports clubs and schools				Study participants recruited from sports clubs (part of the total sample)		
			Female	Male	N/A	Total	Female	Male	Total
2149	619 (mean age 15.5, SD 0.6)	MAINTAINERS	101	87	3	191	75	74	149
		DROPOUTS	167	83	5	255	109	65	174
		NONPARTICIPANTS	112	56	2	170			
		Joiners	2	1	0	3			
		Total	382	227	10	619	184	139	323

### Measures

At baseline, a question battery related to *perceived coach’s health promotion activity on health topics* was used, and the adolescents were asked *‘How often during the past six months has your coach discussed the following health topics with you?’* (see Table 1). The health topics were selected for the battery in a previous study [34]. A 4-point response scale was used: very often, often, rarely, and never. These were dichotomized as *frequent* (very often/often) and *infrequent* (rarely/never). The sum variable, that is, the index of *coach’s general health promotion activity* was formed of the health topics and 33% splitting was used resulting in categories of *frequent, medium,* and *infrequent*. Dichotomization, sum variable and splitting to thirds have also been used in previous studies [14, 34].

The questions related to *lifestyle habits* in follow-up were validated in a previous Health Behavior in School-aged Children (HBSC) study [33, 35]. The same questions were used at both measurement points; hence, based on the recommendation of moderate to vigorous physical activity for adolescents aged ≤ 18 years (60 min/day) [27] *physical activity* was assessed by the following item: *‘During the past seven days, how often have you done physical activity for at least 60 min’* The response options were 0,1,2,3,4,5,6, and 7 days. These were dichotomized as *7 days/week* and *< 7 days/week* to explore whether the recommendation was met.

*Sleep* was assessed using the following items: (i) *‘When do you usually go to bed weekdays?’*, the response options: ‘at 21.00/21.30/…/02.00 a’clock at the latest or later’ (ii) *‘When do you usually wake up weekdays?’*, the response options: ‘At 5.00/5.30/…/8.00 a’clock at latest or later. According to the recommendations, the appropriate sleep duration for young adults (age 18 onwards) is 7–9 h [28]. *Sleep duration* was calculated, and the variable was dichotomized as follows: *≥ 8 h* (average recommendation met) and *< 8 h* (not met).

*The use of salad, fruits, and vegetables* was assessed by the following question: ‘*How often do you eat following food items?*’. The items were ‘salad’, ‘fruits’, and ‘vegetables’. The response options for each item were: ‘never/less than once a week/once a week/2–4 days a week/5–6 days a week/once a day every day/more than once a day every day’. The sum variable was formed and dichotomized as follows: *at least one of these (salad, fruits, or vegetables) more than once a day every day* and *less*, since the recommendation is five portions per day [36].

*The use of cigarette*, *snuff,* and *alcohol* was assessed by the following question: *‘How often do you use cigarette/snuff/alcohol at the moment?’*. The response options for cigarette and snuff use were daily/every week, but not every day/less than once a week/I don’t use’; and for alcohol use ‘Once a week or more often/couple of times per month/about once a month/less frequently/I don’t use’. The variables were dichotomized as follows: *no use* and *use* (= all the other options) since cigarette and snuff impact negatively on health and are a cause of death and disease [37], amount of alcohol used (moderate or binge drinking) was unknown, and no amount of alcohol that is safe for health can be established [38].

*Longitudinal organized sports participation patterns* [19] were used with categories *maintainers* and *dropouts* when the coach’s health promotion activity was explored between the patterns, and with categories *maintainers, dropouts,* and *nonparticipants* when lifestyle habits were explored.

### Statistical analysis

Analyses were performed using SPSS Version 26, with statistical significance set at *p* < 0.05. *Percentage distributions* of female and male sports participants according to how they perceived the coaches’ health promotion activity in different health topics, and of female and male maintainers and dropouts according to how they perceived coaches’ general health promotion activity, and of maintainers, dropouts, and nonparticipants according to the lifestyle habits, were conducted. Due to a small number of males in some categories the lifestyle habits were not analysed separately by gender. *Chi-square test, Fisher’s exact test,* and *z-test* were used to test the differences between organized sports participation patterns. The effect size was estimated using *Cramer’s V. Binary logistic regression analysis* was used to explore how coaches’ general health promotion activity related to organized sports participation patterns, and how the patterns related to lifestyle habits, which, in the bivariate analyses, showed statistically significant differences between the patterns. The models were adjusted for gender.

## Results

The percentage distributions of female and male sports participants at age 15 according to how they *perceived coaches’ health promotion activity in different health topics* are shown in Table 2. The majority of the female and male organized sports participants (51.8–69.6%) perceived coaches’ health promotion activity as frequent in the health topics of sleep and rest, injury prevention, and training when ill.

**Table 2.** The percentage distributions of female and male sports participants at age 15 according to how they perceived coaches’ health promotion activity in different health topics (%).

	Total (*n* = 323)		Females (*n* = 184)		Males (*n* = 139)		χ2			Effect size
Health topics	Frequent	Infrequent	Frequent	Infrequent	Frequent	Infrequent	Value	df	*p*	Cramer’s V
Injury prevention	67.2	32.8	69.6	30.4	64.0	36.0	1.10	1	.294	0.06
Sleep/rest	65.3	34.7	66.3	33.7	64.0	36.0	0.18	1	.671	0.02
Training when ill	56.0	44.0	59.2	40.8	51.8	48.2	1.78	1	.182	0.07
Physically active lifestyle	46.7	53.3	45.7	54.3	48.2	51.8	0.21	1	.649	0.03
Nutrition	46.1	53.9	45.7	54.3	46.8	53.2	0.04	1	.843	0.01
Hygiene	27.6	72.4	29.3	70.7	25.2	74.8	0.69	1	.406	0.05
Cigarette	10.8	89.2	9.2	90.8	12.9	87.1	1.13	1	.288	0.06
Doping substances	10.2	89.9	11.4	88.6	8.6	91.4	0.67	1	.414	0.05
Alcohol	9.6	90.4	8.7	91.3	10.8	89.2	0.40	1	.527	0.04
Snuff	8.7	91.3	6.0	94.0	12.2	87.8	3.91	1	.048	0.11
Violence related to sport	8.4	91.6	7.1	92.9	10.1	89.9	0.93	1	.334	0.05
Drugs	6.8	93.2	5.4	94.6	8.6	91.4	1.28	1	.259	0.06
Sexual issues	2.8	97.2	1.6	98.4	4.3	95.7	2.11	1	.181^F^	0.08

^F^ = Fisher’s exact test.

*Perceived coaches’ general health promotion activity* was associated with changes in organized sports participation among males. Males who had perceived coaches’ general health promotion activity as frequent were more likely maintainers than dropouts (48.6 vs. 20.0%) (χ^2^ (2) = 12.89, *p* = .002, effect size = 0.30). There was no difference among females (Table 3). According to the regression analysis, perceiving coach’s health promotion activity as frequent increased the odds of being a maintainer rather than a dropout (OR 1.78, 95% CI 1.03–3.07, *p* = .038) (Table 4).

**Table 3.** The percentage distributions of female and male maintainers and dropouts according to how they perceived coach’s general health promotion activity (%).

	Females (*n* = 184)		χ^2^			Effect size	Males (*n* = 139)		χ^2^			Effect size
Coach’s general health promotion activity	Maintainers	Dropouts	Value	df	*p*	Cramer’s V	Maintainers	Dropouts	Value	df	*p*	Cramer’s V
Frequent	32.0	28.4	0.37	2	.829	0.05	48.6*	20.0*	12.89	2	.002	0.30
Medium	32.0	35.8					23.0*	41.5*				
Infrequent	36.0	35.8					28.4	38.5				

* difference according to z-test.

**Table 4.** Binary logistic regression analysis of the association of coach’s general health promotion activity with maintained participation compared to dropout (*n* = 323).

	OR (95% CI)	*p*
Coach’s general health promotion activity		
Infrequent	1	
Medium	0.83 (0.48-1.42)	.48
Frequent	1.78 (1.03-3.07)	.038

OR = odds ratio, CI = confidence interval.

The model was adjusted for gender.

The percentage distributions of maintainers, dropouts, and nonparticipants according to the *lifestyle habits* reported at age 19 are presented in Table 5. Maintainers had healthy lifestyle habits more commonly when compared to nonparticipants or dropouts (22.3–93.7 vs. 6.0–74.1 or 6.7–85.9%, respectively), with the exception of alcohol use, when compared to nonparticipants (89.5 vs. 80.6%).

**Table 5.** The percentage distributions of maintainers, dropouts, and nonparticipants according to the lifestyle habits reported at age 19 (%).

Lifestyle habits	Total *n* = 609–616	Maintainers *n* = 188–191	Dropouts *n* = 253–255	Nonparticipants *n* = 168–170	χ2			Effect size
Physical activity					Value	df	*p*	Cramer’s V
7 days/week	11.3	22.3^a^	6.7^b^	6.0^b^	32.88	2	<.001	0.23
< 7 days/week	88.7	77.7^a^	93.3^b^	94.0^b^				
Sleep								
≥ 8 h	66.6	72.8^a^	69.6^b^	55.3^b^	14.05	2	<.001	0.15
< 8 h	33.4	27.2^a^	30.4^b^	44.7^b^				
Salad, fruits, and vegetables								
At least one of these more than once a day every day	33.3	39.8^a^	33.7^a,^^^^^b^^^^	25.3^b^	8.55	2	.014	0.12
Less	66.7	60.2^a^	66.3^a,^^^^^b^^^^	74.7^b^				
Alcohol								
No use	12.5	10.5^a^	9.4^a^	19.4^b^	10.37	2	.006	0.13
Use	87.5	89.5^a^	90.6^a^	80.6^b^				
Cigarette								
No use	85.1	93.7^a^	85.9^b^	74.1^c^	27.43	2	<.001	0.21
Use	14.9	6.3^a^	14.1^b^	25.9^c^				
Snuff								
No use	89.4	90.6^a^	89.0^a^	88.8^a^	0.38	2	.828	0.03
Use	10.6	9.4^a^	11.0^a^	11.2^a^				

a,b,c = differences according to z-test.

In the regression analysis, maintainers had higher odds of meeting the recommendations of physical activity (OR 4.36, 95% CI 2.10–9.04, *p* < .001) and sleep (OR 2.48, 95% CI 1.57–3.92, *p* < .001), eating salads, fruits, and vegetables more than once a day every day (OR 2.25, 95% CI 1.41–3.60, *p* < .001), and not using cigarettes (OR 5.31, 95% CI 2.62–10.73, *p* < .001), but lower odds of not using alcohol (OR 0.49, 95% CI 0.27–0.90, *p* = .021) when compared to nonparticipants. Maintainers had higher odds of meeting the physical activity recommendation (OR 4.05, 95% CI 2.19–7.49, *p* < .001) and not using cigarettes (OR 2.51, 95% CI 1.23–5.10, *p* = .011) when compared to dropouts. Dropouts had higher odds of meeting the sleep recommendation (OR 1.83, 95% CI 1.21–2.77, *p* = .004) and not using cigarettes (OR 2.12, 95% CI 1.28–3.50, *p* = .003), but lower odds of not using alcohol (OR 0.43, 95% CI 0.25–0.77, *p* = .004) when compared to nonparticipants (Table 6).

**Table 6.** Binary logistic regression analysis of the lifestyle habits at age 19 according to the patterns of organized sports participation (*n* = 323).

Organized sports participation patterns	Physical activity 7 d/wk			Sleep ≥ 8 h			Salad, fruits, and vegetables (at least one of these more than once a day every day)			No alcohol use			No cigarette use		
	OR	95% CI	*p*	OR	95% CI	*p*	OR	95% CI	*p*	OR	95% CI	*p*	OR	95% CI	*p*
Maintainers vs. nonparticipants*	4.36	2.10–9.04	<.001	2.48	1.57–3.92	<.001	2.25	1.41–3.60	<.001	0.49	0.27–0.90	.021	5.31	2.62–10.73	<.001
Maintainers vs. dropouts*	4.05	2.19–7.49	<.001	1.36	0.83**–**2.09	.164	1.48	0.99**–**2.23	.057	1.13	0.60**–**2.13	.698	2.51	1.23–5.10	.011
Dropouts vs. nonparticipants*	1.08	0.48**–**2.44	.860	1.83	1.21–2.77	.004	1.52	0.98**–**2.36	.064	0.43	0.25–0.77	.004	2.12	1.28–3.50	.003

OR = odds ratio, CI = confidence interval.

The model was adjusted for gender. *reference category.

## Discussion

This study focused on *sports participants’ perceptions of coaches’ health promotion activity* and how these perceptions predicted *maintained participation and dropout* in organized sports in emerging adulthood. In addition, differences in *lifestyle habits between maintainers, dropouts,* and *nonparticipants* in organized sports were explored.

The majority of the 15-year-old female and male participants in organized sports perceived that *coaches’ health promotion activity in the health topics related to* sleep and rest, injury prevention, and training when ill, was frequent, but in most of the health topics it was infrequent. However, also many sports participants perceived, that coaches discussed injury prevention, sleep, and training when ill, infrequently. These results reinforce previous research on males [14] and adds to the knowledge on females.

It is understandable that health topics related to sports training and performance are frequently addressed during training. However, there may be many reasons why coaches are not active in various health topics. Previous research has shown that lack of time and competence may explain coaches’ inactivity in health promotion [39, 40]. This may especially apply to typical Finnish youth sports clubs, where coaches mainly work on a voluntary basis [32]. In addition, coaches may assume that sports participants behave healthily, for example, are physically active enough and do not use substances, or that other stakeholders like home and school are responsible for adolescents’ health promotion. Moreover, some topics are more sensitive, for example, nutrition and sexual issues, and may be why they are not frequently discussed.

The current study also adds knowledge on gender differences by showing that more likely males than females perceived that snuff use was frequently discussed by coaches. This may be due to more typical snuff use among males which in turn may reflect coaches’ reactive rather than proactive behavior in health promotion as previous study with the same cohort suggests [23].

This study also showed, for the first time, to our knowledge, that among males, those who had *perceived coaches’ general health promotion activity* as frequent were more likely maintainers than dropouts, but among females, there was no difference. A previous study with the same cohort and international research have shown that more commonly males have a strong competitive orientation than females [41, 42]. Hence, one interpretation is that more male maintainers put effort into competitive sports and are participants in sports clubs in which coaches have competence in health promotion and see health promotion activities important for athletes’ success. Among females, other factors such as school studies, injuries, or friends dropping out of sports as significant dropout reasons of the same cohort [19] may influence sports participation, and coaches’ frequent health promotion activity may not necessarily maintain it.

Since there were also many female and male maintainers who had perceived coaches’ general health promotion activity as infrequent, other significant factors for participation in sports, for example enjoyment in sports [43, 44], friends [44], and competitiveness [42], may maintain participation. On the other hand, many female and male dropouts had perceived coaches’ health promotion activity as infrequent. This may affect dropping out, especially if other factors do not favor participation. Moreover, individuals may differ in how they perceive the same quantity and quality of health promotion activities. The quantity and tone of the discussion, especially on sensitive topics such as nutrition in aesthetic or weight-sensitive sports such as gymnastics and figure skating, may affect the intention to drop out.

The current study also added knowledge by showing that maintainers were more likely to have more *healthy lifestyle habits* in emerging adulthood than nonparticipants or dropouts. In addition, dropouts were more likely to have more healthy lifestyle habits than nonparticipants. In previous publications, the 15-year-old sports participants of the same cohort showed to have more often healthy lifestyle habits compared to nonparticipants of the same age [23, 26, 29]. Moreover, another Finnish study showed a positive association between sports participation in adolescence and lifestyle habits in adulthood [25].

Healthier lifestyle habits among sports participants compared to other groups may be due to many factors. Our previous findings suggest for example that perceived parental support for physical activity and sport, adolescents’ academic success [19] as well as competitive orientation [42] are related to maintained participation in organized sports in emerging adulthood. Also, high parental education is associated with participation in organized sports and healthy lifestyle habits among teenagers [22]. Thus, exposure to support and knowledge in various environments i.e. in sports clubs, at school, and at home, and one’s own understanding as well as motivation to behave healthily for success in sports could explain the healthier lifestyle habits of maintainers. On the other hand, more maintainers and dropouts reported alcohol use compared to nonparticipants in the current study; however, at age 15, there was no difference between sports participants and nonparticipants [23]. This result is in line with international research showing that sports participation at teenage years was more likely than nonparticipation related to alcohol use in early adulthood [30]. Friends in sports teams and social pressure may affect alcohol use.

The results of this current study also provide an opportunity to compare the lifestyle habits of those who continued to participate in sports clubs with their lifestyle habits at the age of 15 [26, 29]. The majority of the maintainers in the current study met the average recommendation for sleep of aged 18–25, that is, at least 8 h/night [28], on weekdays, as the majority of the sports participants at age 15 years of the same cohort met this minimum recommendation for sleep of aged 14–17 [28] on weekdays (73% vs. females 78%, males 80%) [26]. However, almost as many maintainers in the current study as sports participants at age 15 did not meet 60 min of physical activity daily (78% vs. 83% f, 70% m) [26] or did not eat salads, fruits, or vegetables many times per day or daily (60% vs. 40–53% f, 66–75% m) [29]. Other activities such as school studies or work together with time used in organized sports training may explain why 60 min of physical activity per day is not met. In addition, it may be usual in emerging adulthood not to think of diseases or losing one’s health, which could explain the low consumption of salads, fruits, and vegetables. These habits are also in line with frequently (sleep) and infrequently (physical activity and nutrition) discussed health topics.

The current study showed some positive results related to perceived coaches’ health promotion activity and maintained sports participation, as well as sports maintainers’ lifestyle habits. However, it also suggests that coaches’ discussions on e.g. injury prevention and many lifestyle habits are not always sufficient or timely, and do not often support maintained participation or promote healthy lifestyle habits since also many maintainers had unhealthy lifestyle habits. These suggestions are also supported by the fact that injuries are common in organized sports [9, 18], and together with a focus on studying, they are a significant reason for dropout among both females and males [19].

Injury prevention strategies used in sports club [17], and coaches’ behavior have been shown to affect the decision to participate in sports [17, 45]. In addition, if there is an injury, it could be important to ensure social connections and involve injured sports participants in the sports society with peers during rehabilitation. Moreover, promoting flexibility in combining studies and sports may support maintained participation in sports. Hence, health promotion may reduce health-related reasons for dropout, such as pressure related to time use or injuries [19, 45]. Furthermore, encouragement of healthy lifestyle habits by coaches has been shown to have a positive effect on participants’ enjoyment of sport [15, 16] which in turn may maintain participation [43, 44].

There is a need to promote coaches’ health promotion activity when it is infrequent, and not forget the responsibility of home and formal education. However, there may also be a need to promote coaches’ knowledge and competence in health promotion [39, 40].

### Strengths and limitations

The strengths of this study include longitudinal data that provides an opportunity to explore longitudinal organized sports participation patterns that are rarely used in studies focusing on coaches’ health promotion activity or sports participants’ lifestyle habits. Moreover, this study explored rarely studied late adolescent age brackets and females.

One limitation is that the perception differences in the quantity and quality of the coaches’ health promotion activity may have affected the questionnaire answers. In addition, for the possibility of getting a trend in the cohort study, the physical activity recommendation for adolescents (i.e. 60 min/day of moderate to vigorous physical activity) was also used at age 19, and not the recommendation for adults (aged 18–64 years; ≥ 150 min/week of moderate to vigorous physical activity) [27]. There might be more study participants who complete the less time-demanding ­physical activity recommendation. Moreover, dichotomization of the variables related to the lifestyle habits hides variation among maintainers, dropouts, and nonparticipants, but provides possibility to explore whether recommendations for lifestyle habits are met. Many measures of lifestyle habits still show a need for health promotion within organized sports activities.

In the future, it is important to study the association of lifestyle habits with sports participation patterns by gender with a larger sample. In addition, measures of quality should be used regards to health promotion activity and lifestyle habits (e.g. related to sleep and diet). Studies using different methods, such as interviews and observations, are also needed to determine the quality of health promotion activities that support maintained participation in organized sports among adolescents.

## Conclusions

Perceiving coaches’ health promotion activity as frequent was related to maintained participation in organized sports among males. Maintainers were more likely to have more healthy lifestyle habits than nonparticipants and dropouts. There is a need to invest in coaches’ health promotion activity when it is infrequent. A more detailed understanding is needed of coaches’ health promotion activity that supports both maintained participation in sports, especially among females, and healthy lifestyle habits in emerging adulthood.

## Data Availability

The data used in this study are not publicly available because they contain identification information. However, some parts of the data may be requested by the principal investigator (SK) upon reasonable request.
